# Miscellaneous

**Published:** 1893-01

**Authors:** 


### MISCELLANEOUS.

### THE LARGEST MILL IN THE WORLD.

Mills are of such various descriptions that it is difficult to compare one description of mill with another. There are flour mills, cotton mills, jute mills, silk mills, rolling mills, &c., &c. As to flour mills, the largest in the world is the Pittsburgh Flour Mill in New York State. That mill converts into flour over 32,000 bushels of wheat per day, and its output would feed two cities as large as New York. The largest production of flour is at Minneapolis, which is a town of mills, and where recently one week's output was 83,200 barrels of flour. The cotton-thread mills at Paisley, Scotland, of J. & P. Coates, Limited, and Clarke's, have of late years assumed gigantic proportions,and employ 10,000 men between them. The largest lumber mill is at Port Blakeley, Washington, the machinery occupying a building 102 feet by 448 feet, and capable of cutting between 500,000 and 600,000 laths per day. In 1890 this mill turned out 68,000,000 feet of lumber, 28,000,000 laths, besides a large amount of other stock. As to rolling mills, the largest are those in connection with Krupp's immense works at Essen, in Germany, which occupy more than 500 acres of land. The largest in England are those at Sheffield and on the Tyne. Lancashire is the great seat of the cotton mills, and among the largest may be mentioned those of Horrocks, Miller & Co., and of Messrs. Calvert, at Preston. The largest cotton mill is stated to be located at Kranholm, Russia. It contains 340,000 spindles and 22,000 looms, requiring 6,000 horse power, and giving employment to 7,000 operatives.

To Prevent Moths.—The most destructive of the household pests is the moth, and the principal requisite for protection against it is promptness and care. The best way to protect garments from the ravages of this busy creature is to wrap them in newspapers, being very careful to leave not even the slighest crack by which a miller may find its way in. This should be done as early in the season as the garments can be spared, and they should be well beaten and brushed before wrapping, in order to dislodge any eggs that may have been already deposited on them. If they are put away late, it is safer to open them sometime during July. The worm will then be hatched, if any eggs had chanced to be left in the garments, and can be seen and killed before it does any damage. Cedar chests are of no more use in keeping out moths than any other tight box. Gum camphor is sometimes put among woolen garments, and tobacco is also used ; but though these may have some effect in keeping the miller away, they are not always safeguards, and the surest way is the simplest, that of wrapping the garments so that nothing can gain an entrance. To keep them out of carpets, sprinkle the floor with turpentine or benzine before laying the carpet, and with a small, flat paint brush apply freely under the surbase and in all cracks. Benzine poured over furniture and carpets where moths are will kill them. Great care should be taken not to use the benzine near a flame of any kind and there should be no flame or fire in the room until the fumes have passed away.—*Demorest's Family Magazine.*

Destruction of Birds.—The many friends of the birds, who have so often and so energetically protested against their use for the adornment of women's hats, will be interested in the fate of the moho, one of the most beautiful of the feathered inhabitants of the Sandwich Islands. These birds are now extinct, and Professor Newton, of Cambridge, England, estimates that not above half-a-dozen stuffed specimens of the species exist in the world.

They were clothed with magnificent yellow feathers, and for the sake of these, which were employed in making robes for the native chiefs of the islands, the birds were relentlessly slaughtered. When the supply became exhausted recourse was had to another yellow feathered bird of the islands, much inferior in beauty, however, and the name O-o, formerly borne by the moho, was transferred to this new victim of savage vanity.

It can hardly be a comforting reflection for those who aid or encourage the slaughter of birds for the adornment of human head-gear, that they are simply imitating a thoughtless custom of the uncivilized natives of a Pacific Island.

The Year of Greatest Growth.—In boys is the seventeenth ; in girls the fourteenth. While girls reach full height in their fifteenth year they acquire full weight at the age of twenty. Boys are stronger than girls from birth to the eleventh year; then girls become superior physically to the seventeenth year, when the tables are again turned and remain so. From November to April, children grow very little and gain no weight ; from April to July, they gain in height but lose in weight, and from July to November they increase greatly in weight but not in height.

